# Coffea Cruda in Enuresis

**Published:** 1892-01

**Authors:** J. C. White

**Affiliations:** Portchester, New York


### COFFEA-CRUDA IN ENURESIS.

J. C. White, M. D., Portchester, New York.

Mrs. W., aged thirty, one child ten months old, has had enuresis three years. Had been treated by several so-called "regulars" during this period. After some experience in treating her, each one in turn had informed her that there was no remedy for her but to have the "bladder forcibly distended with the hot water douche each day, until it was stretched to its normal dimensions. That the walls of the bladder were so thickened and contracted." This trouble had developed gradually and without apparent cause; continued through and subsequent to pregnancy without change of symptom.

She complained of a slight distress in region of bladder; no other symptom but the urgent necessity to void the urine as soon as two or three teaspoonfuls accumulated. The urine was normal. She was well nourished, and in every other respect seemed a perfectly healthy woman.

I was disappointed to find that not Petroleum nor any of the several remedies I used during a period of two years had relieved her in the least.

Rest or position made no difference in her symptoms.

She had had her os uteri lacerated to the extent of an inch in her confinement; the uterus hanging a little lower than normal, but did not infringe upon the bladder; neither was there retroversion.

I, however, operated on the cervix. The repair was perfect, and no change of symptoms.

Eight months after the operation she became pregnant, the vesical irritation continuing throughout the period.

Three days after her confinement she complained of insomnia in such a manner as to suggest Coffea. I placed a dose of the 3d on her tongue; left another to be taken at night, if needed. The effect was charming; said she had not been obliged to rise so often; that she could go two hours without voiding urine.

Two weeks after, the husband called for more of those powders, saying the bladder trouble was returning. I gave him six more powders, 3d, as it was the only preparation I had. The other dose acting so well I desired to test it further.

I have not heard from her trouble since, except that she now goes seven hours without inconvenience and voids as much urine at a time as ever.

She had never complained of insomnia before or any other symptom to suggest Coffea to me.

I mention this case as never in all my reading or clinical experience has this remedy suggested itself to me for urinary trouble. The only symptom in Hering is: "Urine frequent and copious."

Dr. T. F. Allen's *Hand-book of Materia Medica* says: "Pressure upon bladder, obliging him to urinate; frequent urging in morning but micturition scanty and in drops; frequent micturition; burning, tearing in fore part of the urethra. Urine copious about midnight with relaxation of genitals; urine scanty; urine blood red."

Pressure upon the bladder, and later soreness over the vesical region, with urgent desire because of the smallest quantity of urine were the only tangible symptoms in this case. The cure was incidental if not accidental. This experience led me to the relief if not the cure of another case, where the erethism of nervous system was prominent, together with the *pressure upon the bladder.* The latter symptom is more like that of Caladium, I think, than of any other drug.

A lady suffering severely from dysmenorrhœa was promptly relieved by Coffea. The pains were paroxysmal; pressing, bearing-down pains limited to hypogastric region with frequent micturition. The flow was intermittent, profuse, and contained pieces of hard-pressed coagulated fibroid. The neck of the uterus was flexed anteriorily; her menses had been postponed two weeks at this time. Had formerly anticipated pain in back; better by lying. She will require Helonias to cure, Sepia having failed.

Neither of the above cases had the mental anguish or fear of death which text-books point out as a distinguishing feature of the drug. The urine symptoms under Coffea should contain: *Pressing pain with or without soreness over vesical region. Frequent micturition of normal urine; very irritable bladder, can contain but a spoonful of urine without urgent necessity to void it.*

